# Supplementary material for: Spatial genetic diversity and populational differentiation of Ternstroemia sylvatica (Ericales: Pentaphylacaceae) in eastern Mexico
Source: PLoS One. 2025 Dec 26;20(12):e0339000. doi: 10.1371/journal.pone.0339000 (PMC12742792; doi:10.1371/journal.pone.0339000)
Supplement: S1 File — (DOCX) [file pone.0339000.s001.docx]

Supplementary Information S1

**Fig S1** MLG accumulation curve


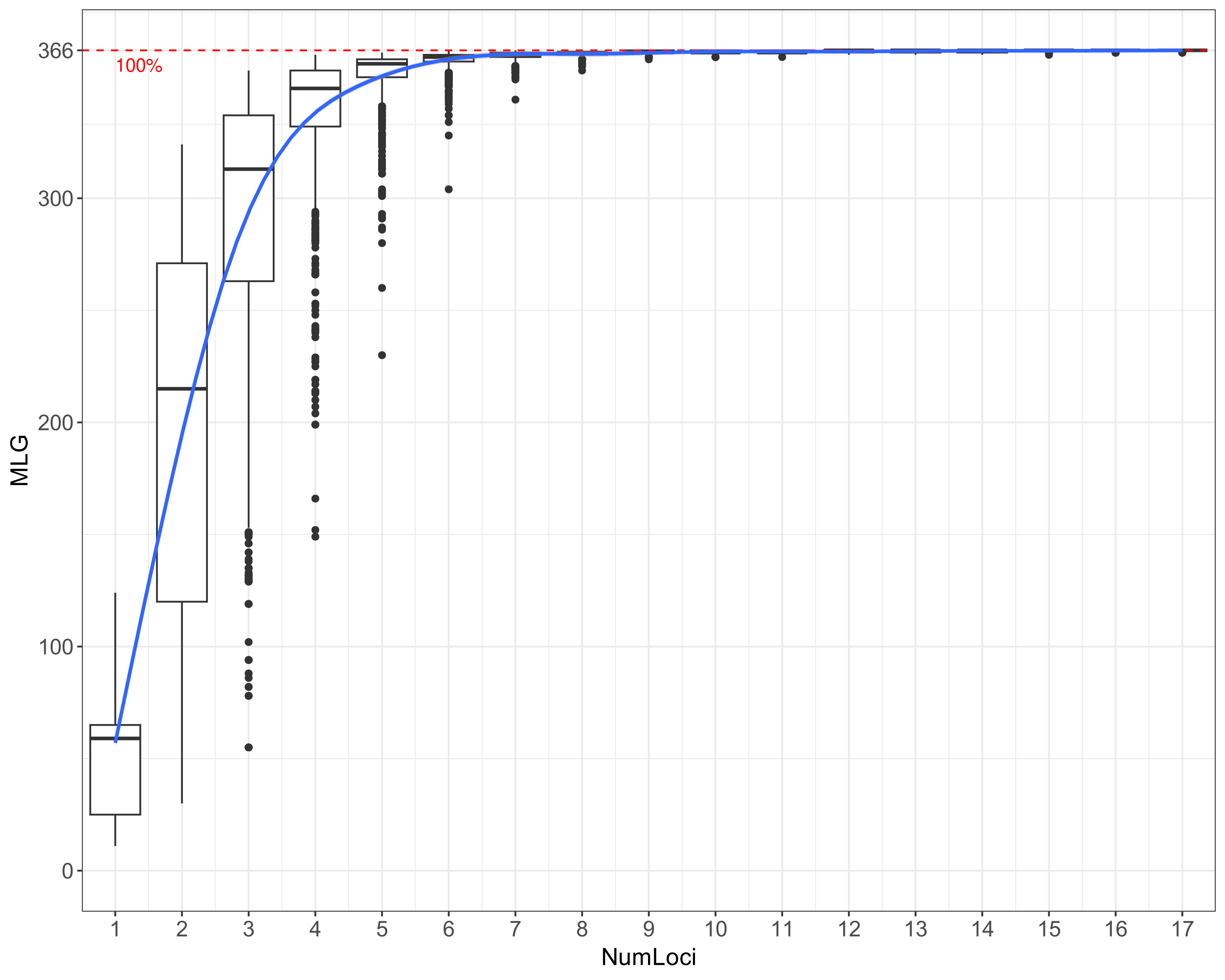


**Fig. S2** Hardy-Weinberg linkage disequilibrium test


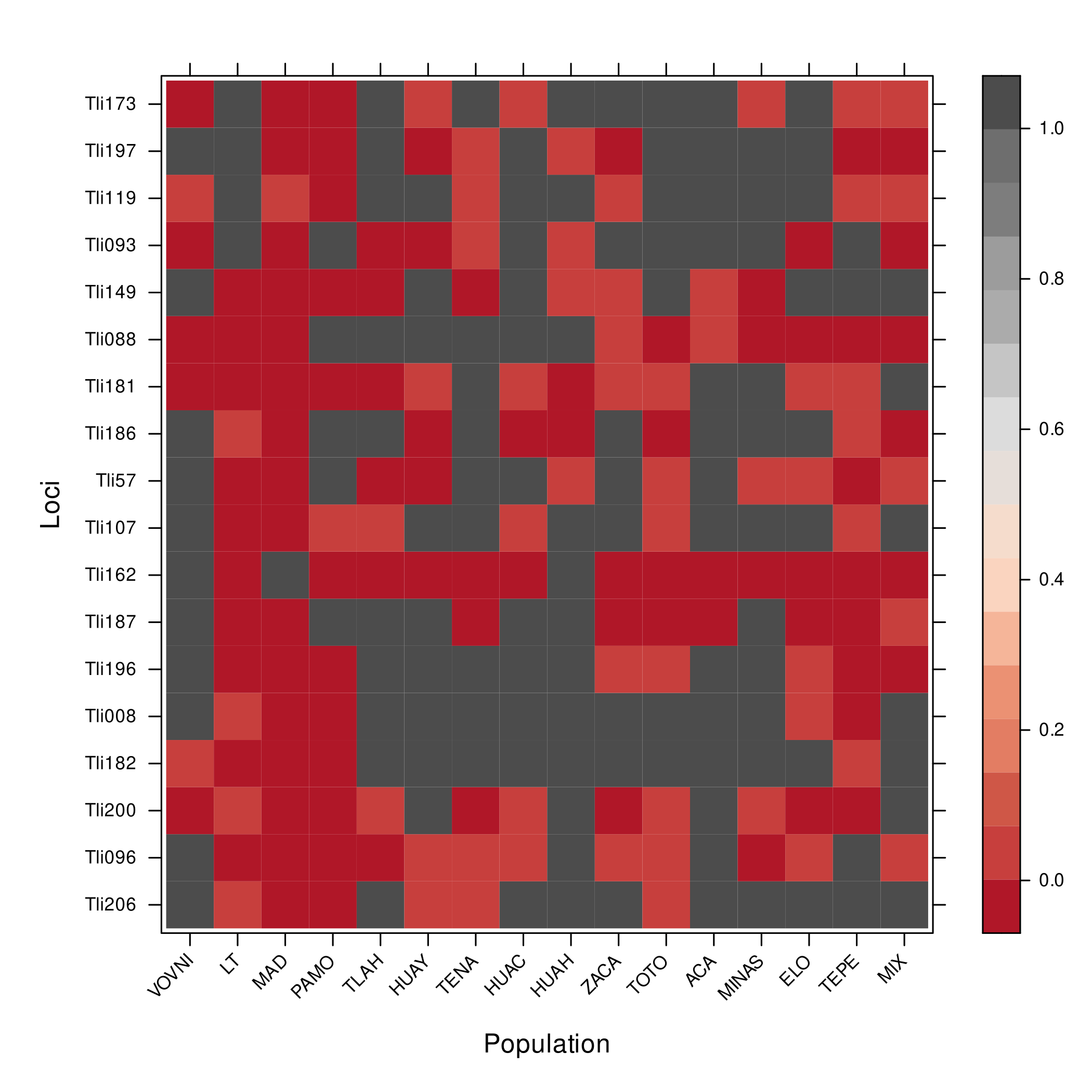


Fig S3
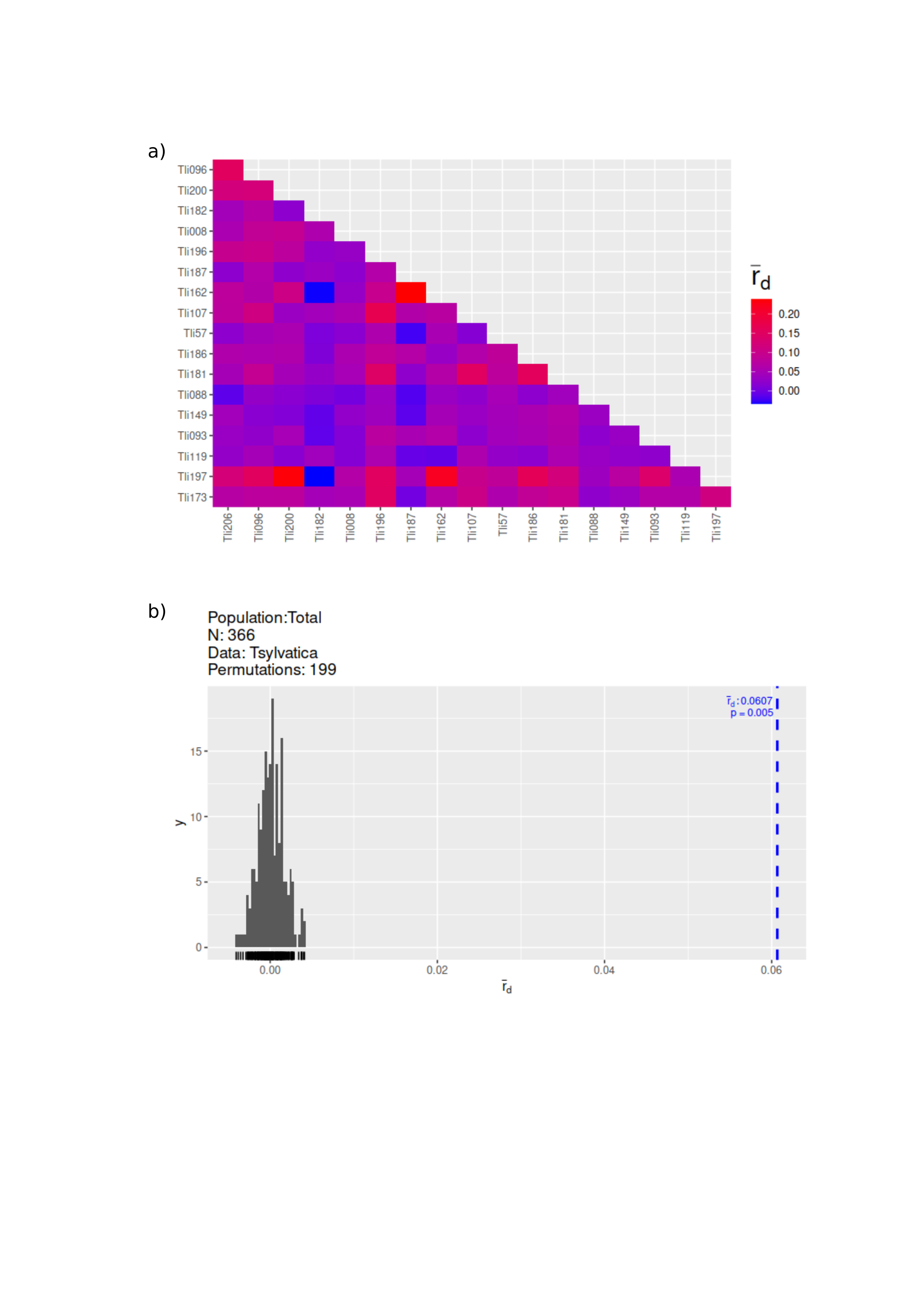
 linkage disequilibrium tests: a) pairwise *r̄_d_* over all loci, b) index of association (*I_A_*) test
