# Supplementary material for: Spatial genetic diversity and populational differentiation of Ternstroemia sylvatica (Ericales: Pentaphylacaceae) in eastern Mexico
Source: PLoS One. 2025 Dec 26;20(12):e0339000. doi: 10.1371/journal.pone.0339000 (PMC12742792; doi:10.1371/journal.pone.0339000)
Supplement: Attachment — Submitted filename: Response to Reviewers.pdf [file pone.0339000.s004.pdf]

July 22nd, 2025.

Dr. Vicente Martínez López
Academic Editor
PLOS ONE

Rebuttal letter on PONE-D-25-08656 Spatial genetic diversity and populational differentiation of *Ternstroemia sylvatica* (Ericales: Pentaphylacaceae) in eastern Mexico

Dear Dr. Martínez:

We appreciate the feedback provided in the recent review of our manuscript. We appreciate the observations and suggestions from both you and the referees as they are positive, relevant, and constructive. Based on the review, we have integrated most suggestions, resulting in a new and improved version. All modifications are introduced using track changes as requested and attached to the resubmission, as well as the formatted final version. Additionally, we are attaching the observations regarding the Journal Requirements, as well as those made by the referees, along with our specific responses to each of them, as part of this letter.

Best regards,

Dr. Gerardo Rivas
Corresponding author

Journal Requirements Response:

1.Please ensure that your manuscript meets PLOS ONE's style requirements, including those for file naming. The PLOS ONE style templates can be found at

R: We have carefully reviewed the PLOS ONE style requirements and, where necessary, applied the journal's guidelines.

2. Please note that funding information should not appear in any section or other areas of your manuscript. We will only publish funding information present in the Funding Statement section of the online submission form. Please remove any funding-related text from the manuscript.

R: We have removed funding information from the manuscript, keeping it only in the funding section of the submission form.

3. In your Methods section, please provide additional information regarding the permits you obtained for the work. Please ensure you have included the full name of the authority that approved the field site access and, if no permits were required, a brief statement explaining why.

R: We have included our collection permission code issued by the Mexican Government.

4. We note that the grant information you provided in the ‘Funding Information’ and ‘Financial Disclosure’ sections do not match.
When you resubmit, please ensure that you provide the correct grant numbers for the awards you received for your study in the ‘Funding Information’ section.

R: We have corrected grant information.

5. We note that Figures 1,5 and 7 in your submission contain map/satellite images which may be copyrighted. All PLOS content is published under the Creative Commons Attribution License (CC BY 4.0), which means that the manuscript, images, and Supporting Information files will be freely available online, and any third party is permitted to access, download, copy, distribute, and use these materials in any way, even commercially, with proper attribution. For these reasons, we cannot publish previously copyrighted maps or satellite images created using proprietary data, such as Google software (Google Maps, Street View, and Earth). For more information, see our copyright guidelines: http://journals.plos.org/plosone/s/licenses-and-copyright.

R: We have replaced the Google Satellite layers in Figures 1,5, and 7 (figures 2, 3, and 7; respectively, in the new version) for a Digital Elevation Model under Open Data Policy, which is equivalent to CC BY 0 license (Public Domain). See terms of use in: https://en.www.inegi.org.mx/inegi/terminos.html. We have also included the reference for this layer.

Responses to Reviewer 1:

Introduction:

The first paragraph is directly describing the species, while to reach a wider audience it could be about the type of question that is going to be explored. For instance, it could be a paragraph about the statement in line 61 '[literature survey showing that] fragmentation affects the genetic diversity and future conservation of tree species'
R: We agree that reorganizing the information in the suggested way will improve the scope and the presentation of the context. We also agree that the suggested mention of fragmentation is pertinent.

Line 60: what does positive mean?
R: We changed the redaction to: these studies demonstrated a positive association between genetic diversity and population size. Thanks for your good suggestion.

Line 61: Maybe spell out the first author's name? e.g., Wehenkel et al. (2017) demonstrated…
R: Done.

Lines 66-67: a bit unclear question. Please try to re-phrase it.
R: We rewrote this line for clarity, making the aims more explicit.

Would commenting/advising about conservation plans for this species be also an aim?
R: We consider that delving into conservation topics is far beyond the aims of this work. However, we are adding some mentions regarding the relevance of describing genetic parameters, such as diversity, rare alleles, and inbreeding index.

Methods:

Is it known that the species is diploid? If no, how do you think this may affect the results?
R: There are only cytogenetic studies on Ternstroemia japonica that report an haploid chromosome (Morinaga & Fukushima, 1931). There aren't specific studies on *T. sylvatica*. Ploidy may be a significant issue for microsatellite analysis when individuals with different ploidy levels are present. However, most R packages and clustering software like STRUCTURE are fully capable of analyzing polyploids. See the following references:

Kamvar, Z. N., Everhart, S. E., & Grünwald, N. J. Data preparation in Population genetics and genomics in R. https://grunwaldlab.github.io/Population_Genetics_in_R/Data_Preparation.html

Pritchard, J. K., Wen, W., & Falush, D. (2003, July). *Documentation for STRUCTURE software: Version 2*. https://citeseerx.ist.psu.edu/document?repid=rep1&type=pdf&doi=e05f0090ca1b0bdd9c198f13250af05548fc2db0

Also, we found no evidence of polyploidy in the microsatellite electropherograms. This can be suspected when more than two alleles show up, but this was not the case (see reference below). Therefore, we are assuming *T. sylvatica* is a diploid. We added a mention of this.

Selkoe, K. A., & Toonen, R. J. (2006). Microsatellites for ecologists: a practical guide to using and evaluating microsatellite markers. *Ecology letters*, *9*(5), 615-629.

Lines 71-72: add dates of collection. How many samples are collected per population? It is interesting that all material is fresh, and no herbarium material is used.
R: We added the collection years. The sample size per population ranged from 15 to 30 individuals, as shown in Table 2. However, considering that this information is relevant to the sampling description, we are repeating it in Table 1.
In previous molecular work with the genus, we have encountered numerous problems using herbarium-preserved specimens. These samples are used to extract a dark precipitate (presumably a mixture of tannins) that hinders DNA isolation and decreases the efficiency of PCR. Additionally, newspaper-preserved specimens consistently result in DNA degradation, leading to a decrease in PCR success.

Line 73: grinded or ground
R: Correction done.

Line 76: *S. sylvatica* should be *T. sylvatica*
R: Correction done.

Line 97: please add the reference after each package name.
R: Citations added.

Line 104: why is K=1 not used?
R: The method proposed by Evanno et al. (2005) is based on the second-order rate of change (second derivative) of the log probability of the data, LnP(D), between successive K values (number of clusters). So, since K=1 has no previous values (there are no values for K=0), ΔK cannot be calculated.

Line 105: there's an extra 'e' after the reference.
R: Correction done.

Line 109: please add reference after QGIS 3.22.4
R: SIG reference added.

Results:

I suggest adding a picture of the species.
R: We consider this to be quite pertinent and illustrative, so we have included a plate of *T. sylvatica* as Fig 1.

Private alleles can be reported for each population.
R: We have included the calculation of private alleles.

Fst value between the northern and southern populations can be reported (based on genetic clustering).
R: This is reported in the "Between region" comparison as F_{CT}. We have made this explicit.

Line 139, 143 and 151: pairwise Fst
R: Correction done.

Discussion:

Do these plants grow vegetatively too? Can it affect the genetic structure? And after how long of fragmentation, genetic effects would be observed?
R: We did not find reports about vegetative reproduction of Mexican *Ternstroemia*. Certainly, the presence of clones can artificially modify the genetic structure, but we were cautious to perform a Multilocus Genotype test that (See supplementary information S1) demonstrated the absence of clonal individuals. Finally, demonstrating the effects of fragmentation on genetic diversity or structure requires at least two generations or cohorts after the event, allowing for the calculation of genetic parameters over two generations. In this case, however, we collected only the reproductive individuals, limiting any temporal/generational inference.

Line 238: an extra 'bitt' after reference.
R: Typing error, corrected.

Line 241: add a space before 'where'
R: Typing error, corrected.

Lines 242-243 gene flow instead of genetic flow
R: Correction done.

Lines 303-end: using private alleles at each population affects conservation action plans. You may consider it.
R: We included the private alleles calculations.

Responses to Reviewer 2:

This study investigated the spatial patterns of population genetic diversity and differentiation of a typical cloud forest tree species *Ternstroemia sylvatica* in eastern Mexico. Using microsatellite markers, the authors estimated genetic indices and applied STRUCTURE and DAPC analysis to identify different genetic clusters. These clusters appear to be explained by geographic separation and local hybridization. Before this study can be published for a broader audience, I have the following thoughts that the authors should consider.
Major comments:

1) **Introduction:**
The goal of this study was not very clearly stated in the introduction. If the primary aim was to present the distribution of population genetic diversity and differentiation of the species, the broader relevance and appeal to a wider scientific audience may be limited. The author should more explicitly articulate the state of the art the study addresses. If the study aims to test the hypothesis that geographical patterns have shaped the genetic structure, e.g. SGSP and MB, this hypothesis should be clearly formulated in the beginning. Additionally, fragmentation is mentioned both in the introduction and later, yet the definition is unclear. What is the difference between fragmentation and biogeographical barrier in the manuscript?

   R: We agree that the use of the term "fragmentation" (when referring to anthropogenically driven fragmentation) may have been misleading or even confused with the "biogeographical discontinuities". We are explicitly integrating the biogeographic hypothesis. Specifically, we are testing the role of the major disjunctions within the distribution. Also, we have summarized the leading causes and effects of human-driven fragmentation as context or justification for this work.

2) **Methodology:**
the AMOVA was not stated in the Methods section. Moreover, the terminology (region, group?) and the interpreted based on this need to be more carefully addressed through out the manuscript.

   R: We have included the AMOVA method, along with the methodological details used to conduct it. Additionally, we have standardized the use of hierarchical terms to avoid confusion, especially when it comes to variance sources like regions, populations, and individuals; instead of the generic and often misleading term "samples" generated in the package *poppr* by default.

3) **Interpretation of the results:**
When interpreting the results, the manuscript would benefit from including statistical analysis and adding references or comparisons with other studies or species. If no studies were found, theoretical work should provide a frame. This would help to substantiate the claims made based on the observed values (see below for specific points). Several studies were briefly mentioned in the introduction, however without relationship to the result of the current study.

   R: We revised the Discussion section to clearly explain what the statistical results mean in terms of population structure and genetic differentiation.

Although these analyses (e.g., Hardy-Weinberg tests, AMOVA/F-statistics, DAPC, STRUCTURE) were already presented in detail in the Results section, we now highlight their relevance more clearly by interpreting their implications for population structure and genetic differentiation. We also draw comparisons with findings from previous studies to substantiate our conclusions and provide a stronger contextual framework for our results.

In parallel, we added new references and re-evaluated those already mentioned in the Introduction, ensuring they are meaningfully integrated into the Discussion. These additions help position our results within the broader literature and enhance the clarity and impact of the study's contributions. We believe these revisions significantly improve the manuscript and address the reviewer's thoughtful suggestions.

4) **Discussion structure:**
The structure of the discussion should be improved. Under the first subtitle, the content is not well structured. While the second and last paragraph contain some repetition to the content in the first subtitle and partly disconnected.

R: We have generated a proposal to restructure the discussion so that it is clearer and reduces repetition. This allows the information to be read from a general perspective to a specific one.

5) **Conclusion:**
After reading the manuscript, I find the conclusion that that species exhibits generally general high genetic differentiation is not fully supported. Most of the genetic variance is found within population not between them. The interpretation of inbreeding depression should be first properly addressed in discussion by comparison with other studies. The last paragraph regarding conservation implementation feels disconnected and relies heavily on general statements without sufficient support from the findings. I recommend that the authors focus more closely on summarizing the main results and their direct implications, and avoid broader claims that are not firmly grounded in the data.

R: We appreciate the reviewer's comments on the conclusion and acknowledge the importance of ensuring that our interpretations and recommendations are firmly grounded in the data. Below, we addressed each point in detail:

1. **On genetic differentiation:**
We respectfully disagree with the notion that high genetic differentiation is not supported. While it is true that most genetic variation resides within populations (see references below in point 2)—a common pattern in outcrossing, long-lived plant species, like seems to be the case in *Ternstroemia*—the observed F_{ST} values in our study are within the range typically interpreted as moderate to high genetic differentiation (e.g., Wright 1978; Hartl & Clark 1997). To strengthen this conclusion, we have now included additional examples from the literature

reporting similar F_{ST} values in comparable species, reinforcing the biological significance of our findings.

2. **On within-population variation and broader patterns:**
   We fully agree that forest tree species, particularly those in temperate ecosystems, often show high within-population genetic diversity. We have clarified this in the Discussion and contextualized our results by referencing foundational studies such as:

   Loveless, M. D., & Hamrick, J. L. (1984). Ecological determinants of genetic structure in plant populations. Annual review of ecology and systematics, 65-95.

   Hamrick, J. (1989). The genetic structure of tropical tree population: Associations with reproductive biology. *The Evolutionary Ecology of Plants*, 129-146.

   Hamrick, J. L., Godt, M. J. W., & Murawski, D. A. and Loveless, M.D. (1991). *Genetics and conservation of rare plants*, 75.

   The above cited references describe how life history traits and mating systems, shape genetic structure in plants. These references help reconcile our finding of substantial within-population variance with the evidence of overall differentiation between populations.

3. **On inbreeding depression:**
   We have revised the Discussion to better substantiate our interpretation regarding inbreeding depression. We now reference studies that have documented inbreeding effects in forest tree species, providing comparative context and supporting our inference based on observed heterozygosity and F_{ST} values.

4. **On conservation and the final paragraph:**
   We agree with the reviewer that the previous version of the conservation section was too general. We have now revised the conclusion to focus more closely on the specific implications of our findings for conservation, avoiding broad or unsupported claims. The final paragraph now more clearly summarizes key results and ties them directly to conservation strategies relevant to the species studied.

We believe these revisions have improved the clarity, rigour, and impact of our conclusions, and we thank the reviewer for their constructive critique.

Minor points:
Line 41: „land change“, do you mean landscape/land-use change? historical climate change is not “land change”.
R: Thank you for pointing this out. We meant land-use change, especially human-driven transformations in the Sierra Madre Oriental and other tropical montane forests

in Mexico (such as deforestation, agriculture, and urban expansion). We have now corrected the wording in the manuscript to reflect this more accurately.

Line 43: this sentence seems very abrupt and misplaced.
R: We have included this idea within the anthropogenic processes against *T. sylvatica* populations.

Line 60: incomplete sentence. A positive relationship between genetic diversity and population size?
R: Correct, the term "association" or "correlation" was missing.

Line 61: please be more specific what kind of fragmentation effects are referred to?
R: We have revised the sentence to specify the fragmentation causes and effects we refer to, including "habitat isolation, reduced gene flow between populations, and increased edge effects, all of which may influence the genetic structure of the species". This clarification has been included in the revised manuscript.

Line 62: "Following this author..." is unclear. It would be helpful if the author explicitly stated the main message or findings of the cited publication. Additionally, the knowledge gap that motivated the current study should be clearly articulated in relation to the previous work. This would help the reader better understand the rationale and significance of the study.
R: We consider this suggestion highly pertinent. Consequently, we have exposed the knowledge gap by summarizing the problems uncovered in the introduction.

Line 76: two populations are missing in the Fig 1
R: All the populations are displayed, but unfortunately, in close populations, the labels would be so close that the Geographic Information System fails at representing them. We have corrected this issue.

Line 105: "e" should be deleted
R: Correct, typing error. Change applied.

Line 118: the acronym "VOVNI"/"OVNI" is not consistent across the manuscript. Please check.
R: We have corrected this discrepancy throughout the manuscript.

Line 136: the AMOVA was not mentioned in the Methods.
R: We have included the AMOVA details and the considered hierarchical levels as indicated in the general observations of the Method section.

Line 152: "n" should be "N"; "number fo individuals" should be "number of individuals
R: We will stick to biostatistics practice, where "N" stands for the whole sample analysed and "n" for each sample (in this case, each population). "fo" was replaced by "of".

Table 1: the number of sampled individuals in each population is very different. And what about the (estimated) population size? This could be a source for the difference in genetic measures, especially allelic richness. Please justify.

Thank you for this critical observation. The number of individuals sampled per population reflects the availability of mature individuals in each forest patch accessible during fieldwork. We acknowledge that differences in sample size can affect diversity estimates, particularly allelic richness. To account for this, we included rarefied allelic richness, which standardizes richness estimates across populations with unequal sample sizes, thereby allowing for meaningful comparisons.
Although we had initially considered presenting only raw allelic richness— as we had used these same markers in a previous study— our main objective here was to assess how marker performance translates in a cross-amplification context. Nonetheless, to avoid bias and better support our conclusions, we relied on Expected Heterozygosity (*He*) as the primary measure for comparing genetic diversity among populations.

Table 3: The terms "region" and "samples" are unclear. Please define them clearly. Also, the AMOVA should be fully described in the Methods section.
Thank you for the observation. We have now clarified in the Method section how "regions" were defined, based on the geographical structure of populations, and what is meant by "samples" in the context of the AMOVA (i.e., individual genotypes within populations). Additionally, we have included the description of the AMOVA analysis in the Methods section. We have made explicit how regions are defined and changed what samples refer to in the other variance sources.
Table 3: please align the columns in the table.
We appreciate the suggestion. All contents in Table 3 have now been left-aligned to ensure consistency with the formatting used in the other tables.

Line 182: "MINAS + ACA" should be "MINAS + ELO"?
R: Thank you for catching this. You are correct—this was an oversight. The group in question should indeed be "MINAS + ELO," and the correction has been made accordingly in the text.

Line 208: All numbers should be reported with two decimal places to align with the formatting used in Table 2.
R: We have applied consistent formatting and now report all numerical values with two decimal places, in line with Table 2.

Line 207-Line 208: please provide a statistic test (e.g. t-test) to support the claim that "the northern group has a lower expected heterozygosity (He) than the southern group".
We conducted a t-test comparing the expected heterozygosity (*He*) between the northern and southern groups. The results of this test have been added to the Results section. See lines 139 and 164-165.

Line 210: The interpretation of "genetic erosion" based simply on the He value is too abstract. Suggest providing additional evidence or set in comparison of other studies.
R: We appreciate this valuable comment. To strengthen our interpretation, we have now included references to comparable studies where reductions in expected heterozygosity have been associated with genetic erosion in forest tree species. This comparison helps contextualize our findings and supports the inference made. See lines 248-249.

Line 210-212: Figure 7 and related text should be put into Results section.
R: We agree with this suggestion. Therefore, we included the genetic diversity values across the space in the Results section and re-labelled the figures accordingly.

Line 218: what is "PAE" analysis?
R: Parsimony Analysis of Endemism (PAE) is a biogeographic method formerly used to identify areas of endemism. We included the full name.

Line 225: The phrase "values of He about Ho" is unclear.
R: We agree, the phrasing was unclear. We have revised the sentence to enhance clarity and accurately reflect the comparison between expected heterozygosity (*He*) and observed heterozygosity (*Ho*).

Line 225: are the values of Fis of 0.42 and 0.38 considered very high? The authors should provide some references or a theoretical framework to make the interpretation of "inbreeding depression".
R: Thank you for this important observation. Although Wright's inbreeding coefficient (F_{IS}) ranges from -1 to 1, its interpretation depends on the reproductive biology and life history of the species. Values above 0.3 are often considered high in outcrossing, long-lived species such as forest trees. To support our interpretation, we have added references to studies on tropical montane forest species where similar F_{IS} values were associated with inbreeding or limited gene flow. These citations help provide a proper context for our discussion of potential inbreeding depression.

Line 233: is Fst=0.205 very high? The author should contextualize this result by comparing it with other studies. Additionally, the logic of the paragraph needs clarification. If the argument is that insect pollination should result in a lower genetic differentiation, it should be noted that the whole studied area covered hundreds of kilometres, which is likely beyond typical insect dispersal distances, if not stated specially.
R: It is high/great, not very high (>0.25). The index was defined by Wright (1978), ranging from 0 (no differentiation) to 1 (total differentiation). However, the qualitative intervals (little/low, moderate, great/high, very great/very high) are based on empirical observations and continue to be used as reference (Hartl & Clarck, 1997). We are replacing other similar terms (but also used in literature) such as low, high or very high to avoid confusion. We are including some references of temperate forest species within the same intervals.
We consider that the previous redaction could be misleading. We have performed some adjustments to point out that there is considerable variation within populations. Thus, suggesting outcrossing by insect pollinators. We agree with the statement that insect pollination is not compatible with long-distance gene flow.

Line 234: It is unclear what is meant by "two main regions." Please specify which regions are being referred to.
R: It referred to the genetic groups instead. We removed this sentence along with a new redaction as indicated in the previous point.

Line 238: "bitt" should be removed.
R: We agree. Removed.

Line 241-243: the word “species” is missing after “TCMF”. It is also unclear whether a high among-population structure is expected? If so, how high and why? This was not so clear to me. And what does the author mean by “ecological niche”? pollination? Seed dispersal? Local adaptation? Micro-climate? Specific definition is needed.
R: We included the word species. We expected very high genetic differentiation because some *Magnolia* species co-distribute with *T. sylvatica* in the Sierra Madre Oriental. *Magnolia* is one of the most widely distributed genera in this region that has been studied in terms of population genetics studies. So, it is a natural candidate to discuss our data with. We include brief insights and/or examples for each term, but we do not consider it necessary to delve into each topic. Regarding local adaptation and microclimate, we do not assume there are sufficient elements to discuss, both due to the supposedly neutral nature of microsatellite markers and the local availability of comparable studies on these topics.

Line 246-247: was the region differentiation applied between “northern group” and “southern group”? then why would MB gap explain this? MB gap is within the northern group, isn’t it?
R: You are correct—the MB gap lies within the northern group but does not fully coincide with the division between the northern and southern groups. Our previous wording was misleading. The MB gap may help explain the substructure observed within the northern group, but it is not the main geographic feature driving the overall north–south differentiation. We have corrected this section in the manuscript to reflect this more accurately and to avoid confusion.

Line 248: what is “first-level genetic structure”? K=2? please be consistent
R: We appreciate the reviewer’s suggestion, though we would like to clarify that the terminology we use reflects standard and widely accepted practices in population genetics when applying the STRUCTURE software. Specifically, it is common to perform STRUCTURE analyses in a hierarchical framework, as established in Vähä et al. (2007), where the first level of genetic structure identifies major divisions, and subsequent levels explore substructure within those clusters.
In our study, the first level of genetic structure is represented by *K=2*, as supported by the Evanno method. This first level reflects the most prominent genetic division among populations, and we consistently refer to it throughout the manuscript using the term “first level genetic structure (*K*=2)” to avoid ambiguity when discussing deeper hierarchical patterns.
This hierarchical interpretation has been widely adopted in recent literature:

Zhang, C., Tang, Y., Tian, D., Huang, Y., Yang, G., Nan, P., ... & Zhang, W. (2022). Population genetic structure of Wikstroemia monnula highlights the necessity and feasibility of hierarchical analysis for a highly differentiated species. *Frontiers in Plant Science*, *13*, 962364.

Wang, Z. M., Meng, S. Y., & Rao, G. Y. (2019). Quaternary climate change and habitat preference shaped the genetic differentiation and phylogeography of Rhodiola sect. Prainia in the southern Qinghai–Tibetan Plateau. *Ecology and Evolution*, *9*(14), 8305-8319.

Ghedina, A., Galla, G., Cadalen, T., Hilbert, J. L., Caenazzo, S. T., & Barcaccia, G. (2015). A method for genotyping elite breeding stocks of leaf chicory (Cichorium intybus L.) by assaying mapped microsatellite marker loci. *BMC Research Notes*, *8*, 1-12.,

and our terminology is consistent with these approaches. We have also clarified this in the manuscript to ensure coherence and to pre-empt any potential misunderstanding regarding our use of STRUCTURE results.

Line 259: "first-level genetic structure" see comment above.
R: See above for the detailed explanation.

Line 260-262: this part of interpretation could be integrated into Line 228-229.and what does the region here specifically refer to?
R: Lines 228-229 refer to genetic diversity, and lines 260-262 refer to genetic clustering. So, we consider it is not repetitive but may represent two different pieces of evidence for the same phenomenon: hybridization. Therefore, we have modified the redaction to make the sections support each other in relation to the hybridization. Additionally, we included a mention of the region where this may occur.

Line 266-267" what does the author mean "increasing fragmentation"? what does fragmentation refer to?
R: We consider that rating (as increasing) current forest fragmentation in eastern Mexico could be a bit ambitious, as it would require support with concise data on forest area loss. As stated in observation L. 61 and explained in the MS, the forests in eastern Mexico have undergone severe historical forest fragmentation due to land-use changes and logging. Therefore, we must consider the current forests in the region as a set of fragments. See:

Ponce-Reyes, R., Reynoso-Rosales, V. H., Watson, J. E., VanDerWal, J., Fuller, R. A., Pressey, R. L., & Possingham, H. P. (2012). Vulnerability of cloud forest reserves in Mexico to climate change. *Nature climate change*, *2*(6), 448-452.

Leija-Loredo, E. G., Pavón, N. P., Sánchez-González, A., Rodriguez-Laguna, R., & Ángeles-Pérez, G. (2018). Land cover change and carbon stores in a tropical montane cloud forest in the Sierra Madre Oriental, Mexico. *Journal of Mountain Science*, *15*(10), 2136-2147.

Line 271-Line 277: this paragraph is not relevant to the current manuscript.
R: We agree that this section may be too specialized and based mainly on unpublished data which could make the manuscript difficult to read. Therefore, we have decided to remove this section.

Line 278-284: this paragraph could be placed after Line 251.both discussing biogeographic barrier.
R: We integrated the biogeographic discussion within the genetic diversity and differentiation, as indicated above.
