# Supplementary material for: Spatial genetic diversity and populational differentiation of Ternstroemia sylvatica (Ericales: Pentaphylacaceae) in eastern Mexico
Source: PLoS One. 2025 Dec 26;20(12):e0339000. doi: 10.1371/journal.pone.0339000 (PMC12742792; doi:10.1371/journal.pone.0339000)
Supplement: Attachment — Submitted filename: Rebuttal .pdf [file pone.0339000.s006.pdf]

Rebuttal letter on PONE-D-25-08656 Spatial genetic diversity and populational differentiation of *Ternstroemia sylvatica* (Ericales: Pentaphylacaceae) in eastern Mexico

October 22, 2025.

Dr. Vicente Martínez López
Academic Editor, PLOS ONE

Dear Dr. Martínez López:

We thank you and the reviewers for the careful evaluation of our manuscript. We have revised the paper thoroughly in response to the new round of comments. Below, we provide a point-by-point response indicating exactly what we changed and where. We are submitting the following updated files: (1) 'Revised Manuscript with Track Changes', (2) a clean 'Manuscript', (3) this 'Rebuttal letter' letter including the response to reviewers, (4) updated figures (with an acronym correction), and (5) updated authors' affiliations. We have also ensured that the discussion uses precise terminology and avoids over-generalization.

We provide a summary of key revisions in this round:

- Categorical labels based on generic thresholds were removed.
- Terminology tightened throughout: we avoid overstatements and replace "genetic erosion" and "inbreeding depression" with precise, data-supported wording.
- Comparisons are restricted to methodologically comparable studies, with explicit caveats when not fully comparable.
- Discussion restructured around three messages: (1) pattern of diversity; (2) spatial structure/biogeographic break; (3) conservation implications.
- Abstract updated accordingly; figures and tables corrected and standardized (including subregional context, acronyms, and decimals).

In relation to Journal Requirements:

- We have no specific citation recommendations to request.
- The reference list has been checked carefully.
- Because part of the Discussion was restructured, the reference list was renumbered and pruned as follows: former Reference 43 was removed; former 44 is now 43; former 45 is now 44; former 48 was removed. All in-text citations and the reference list have been updated accordingly.

Following this section, you will find our point-by-point response to the reviewers. We hope these changes fully address the remaining concerns. We thank the reviewers for their constructive input and remain at your disposal for any further clarifications.

Sincerely,

Dr. Gerardo Rivas (Corresponding author)

Reviewer #1: General comments: The manuscript has undergone substantial improvement and the overall message and aims of the study are now clearer. However, before it can be considered for publication, the main concern remains the first aim: evaluating genetic diversity and differentiation. I strongly encourage the authors to remain closely aligned with their data and to use appropriately comparable studies to support their statements. Please avoid overstatements and ensure precise and careful use of terms such as “genetic erosion”, “inbreeding depression”.

The structure of the discussion has improved considerably, but I recommend further effort to reconsider the placement of some content, organizing it around the main messages rather than the workflow, while also avoiding repetition.

Please also adjust the abstract accordingly.

Specific points are detailed below.

R. We appreciate the reviewer’s positive feedback and take the remaining concerns seriously. In the revision, we (1) strictly align Aim 1 with our own estimates (e.g., F_{ST}, AMOVA) and remove categorical labels from generic thresholds; any cross-taxon numbers are used only as numerical orientation with explicit caveats; (2) remove overstatements and tighten terminology throughout—replacing “genetic erosion” and “inbreeding depression” with precise, data-supported language; (3) limit comparisons to studies that are methodologically comparable and state limitations where they differ; (4) restructure the Discussion around the main messages (pattern of diversity; spatial structure/biogeographic break; conservation implications), relocating or removing repetitive content; and (5) adjust the Abstract according to these changes. We also improved readability by adding subregional context to the map and Table 1, standardizing reporting, and clarifying methods where requested.

Line 49: The region of Sierra Gorda may need some explanation. I suggest providing a map (or adding to the existing map) of the relevant geographical and/or administrative units and adding this information in the Table 1 for each sampled population. It would greatly improve readability for readers unfamiliar with the region, particularly in the later sections when the relationship between population genetic pattern and geographical pattern is discussed.

R: We appreciate the thoughtful suggestion from the reviewer. In fact, subregionalization is an aspect we overlooked because of our familiarity with the area. We have updated Figure 2 to display the referenced subregions and added regionalization and subregionalization to Table 1. We believe this information can offer a better spatial context for potential readers.

Line 56: The meaning of “have not been evaluated” is unclear. Please clarify what the author intends to convey here? Dose this imply that the aspect is highly relevant but has not yet been assessed, or that it is not relevant in this context.

R: Certainly, as the reviewer pointed out, this sentence was unclear: we aimed to highlight the lack of assessment of the effects of harvesting on *T. sylvatica* as a knowledge gap. We have added a clear sentence indicating the absence of studies on species sustainability related to extraction.

Lines 65–70: This sentence is overly long and dense, making it difficult to follow. I suggest breaking it into two or more sentences and clarifying the main points. In addition, the broad generalization (“have demonstrated isolation by distance…, low to moderate genetic diversity…”) may be problematic, as not all of the cited studies necessarily report all of these patterns. Please ensure that the summary accurately reflects the findings of each reference.

R: Thank you for this constructive comment. We agree that the sentence was too long and that our wording could be interpreted as a broad generalization. We have split the section into shorter sentences and softened the claims so that the appropriate subset of studies supports each point. In the revision, we state that regional spatial genetic structure and limited gene flow are commonly reported (e.g., 5,7–9,12), and that several studies examining disturbance/demography report a positive association between population size and genetic diversity (11,13,14). We believe this revised text more accurately reflects the findings of the cited literature.

Line 74: “resulting” should be “resulted”

R: We have applied this correction.

Line 75: The phrase “land-change use to …” is unclear. Suggest revising to “land-use change due to agriculture and cattle raising” for clarity.

R: We have applied for this correction.

Line 81-82: “Moreover, the genus….of its genetic variation” is repetitive with the Line 79-80: “the lack of knowledge… genetic diversity”. Suggest merging or removing.

R: We agree that the sentence is repetitive; therefore, we removed it.

Table 1: The Acronym “Tepe” should be “TEPE”.

R: We have corrected the acronym to use uppercase letters.

Line 129: The settings of the DAPC should be reported in detail. Please specify the discrimination functions and the number of principal components used.

Thank you for the suggestion. We have added the DAPC settings to the Methods (lines 1137–1147 now): we retained 29 principal components (selected by alpha-score optimization) and 4 discriminant functions (K = 5 groups; therefore $K - 1 = 4$).

Line 132: The methods state that the definitions of “northern group” and “southern group” are based on the first-level STRUCUTRE analysis, but the details of which populations are included are provided at the end of the result. This makes the results difficult to follow. Suggest adding this information already here or at least proving a clear reference to where it is given.

R: We agree that the lack of context regarding the correspondence between populations and the first-level genetic groups might confuse the readers. Therefore, we have updated this section to include the population IDs of both groups.

Line 142: “Poliploidy” should be “polyploidy”.

R: We made this correction.

Line 163: “higher” should be “lower”. In addition, why there are no comparisons of Ho and Ar (AR)? If both measures have been reported and the intention is to compare genetic diversity between groups, or state that the genetic diversity is generally lower in the north than in the south, why is only He considered? Suggest either omitting Ar and Ho or state the rationale

R: We have replaced 'lower' with 'higher' because it was mistakenly stated. We considered Expected heterozygosity (He) since it is generally regarded as a more robust and informative measure of genetic diversity than observed heterozygosity (Ho) or allelic richness (Ar). This is due to its statistical properties and biological interpretation: it is less biased by sample size, incorporates allele frequency distributions, and provides a comparable and biologically meaningful estimate of genetic diversity. Instead of including the previous explanation, it is sufficient to focus on the comparisons of He as suggested.

Figure 2, 3 and 4: Acronym “OVNI” should be “VOVNI”

R: This acronym has been corrected in all the figures.

Line 201: “hereafter referred to as the northern group” is incorrect as “northern group” was already used before in Methods and Results. Suggest change to “referred to as “northern group” throughout the study”. The same applies to “southern group”. See also comment at Line 132.

R: As the reviewer rightly points out, we already defined these groups in the methods section, so we agree that it is unnecessary to specify "hereafter" and we have applied the suggestion.

Line 225: “MAD” should be “PAMO” I assume.

R: We reviewed the individual DAPC assignment, and it is correct: cluster number 3 (yellow) contains individuals from VOVNI and MAD, not PAMO. However, the rest of MAD individuals belong to cluster number 5 (blue), hence the duplication of the MAD label among the clusters.

Line 241: Please clarify why two numbers are reported when only one species is considered—are these values derived from different studies? In addition, the corresponding reference is missing and should be provided.

R: Early versions of the manuscript cited values from a published work on *T. lineata* and from unpublished research on *T. huasteca*. However, according to PLOS ONE policies, unpublished data requires including the entire manuscript as a supplementary file. We considered that this would complicate things just to support a single value, so we decided to omit it. However, we forgot to remove the *T. huasteca* value. We left only the *T. lineata* published value.

Line 241-Line 243: For readability, consider combining into a single sentence. For example: “It is comparable to T. lineate (He=…), which is similar in ecological terms (temperate forest dwellers), has similar fragmentation issues and was evaluated using the same microsatellite set.” However, note that T. sylvatica was stated as a tropical instead of temperate species (Line 41), so this statement may need revision.

R: We appreciate the reviewer’s suggestion and have made changes to improve readability. Additionally, we must admit that although *T. sylvatica* is found in Tropical Montane Cloud Forest, it is not exclusive to

this vegetation type and also occurs in some temperate forest ecosystems. Therefore, we have updated this in the abstract and Introduction.

Line 245: Please standardize the reporting of values to two decimal places throughout the manuscript.

R: We rounded all decimal numbers throughout the manuscript. However, the X and Y coordinates in Table 1 were not rounded, because two decimal places do not help locate populations on a map. Conversely, the populations projected on the map in Fig. 2 could potentially eliminate the need to keep X and Y data in Table 1.

Line 249: The reported He of A. guatemalensis is below 0.1, whereas the He of Magnolia species was interpreted as media to high. In pine, He is mostly related to population size but not isolation. Therefore, the comparison with the cited studies may not be appropriate. Consequently, the conclusion that T. sylvatica suffers from genetic erosion is not fully supported and should be revised. Also, the explanation the low diversity using attribution to population isolation at this place is arbitrary.

Nevertheless, the small population size in the current study, only about 20 individuals per population and all available adults were sampled, could be a good explanation for the low He, if the author insists to interpret as such.

R: We acknowledge this point and agree that cross-species comparisons of *He* can be problematic due to differences in demographic history, mating systems, marker sets, and analytical criteria. Therefore, we removed those comparisons as direct evidence and reframed our interpretation to emphasize the intraspecific pattern.

Line 250-260: The MB gap falls within the region of “northern group”. If the authors wish to state this subdivision, other clearly defined terms other than “northern, low-diversity group” and “southern, high-diversity group” should be used as this conflict with the defined terms in Line 201-202. One idea could be to report this subdivision in the results (Line 222-Line 228).

R: Thank you for this helpful suggestion. We agree that our use of “northern, low-diversity group” and “southern, high-diversity group” was ambiguous. We have revised the text to separately reference the biogeographic disjunction of the Moctezuma Basin (MB) and the “northern/southern genetic groups” defined by the first-level genetic clustering. Following your recommendation, we described the “northern group” subdivision in relation to the manuscript, reporting the *He* values associated with each side of the MB. In the revised text, we specify that populations north of the MB include VOVNI, LT, MAD, and PAMO (with *He* = 0.53–0.60), while populations south of the MB show moderate to high diversity (up to *He* = 0.73). We explicitly link these patterns to Figure 3 (interpolated *He*) and note that the distributional break across the MB shown in Figure 2 corresponds to the transition in genetic diversity. Finally, to avoid introducing new terminology, we removed the terms “low-diversity group” and “high-diversity group”. We hope this resolves the inconsistency and improves clarity.

Hidalgo should be at least shown on the map so that the referred populations are clear (see also the comment at Line 49). In addition, the content here seems misplaced. I suggest moving this section together with Lines 293-307, as both address this biogeographical pattern.

R: Since Hidalgo is a political boundary, it is irrelevant to biogeographical discussion. Therefore, we replaced the Hidalgo region we intended to refer to with the term "southern SMOr," which we already introduced earlier. However, in biogeographical terms, the coincidence with the quote above is sufficiently relevant to keep this section.

Line 264: “notorious” change to “noteworthy”

R: We made the change.

Line 264: Specify which measure was considered for this statement “high diversity spot”.

R: We are now specifying that this refers to *He*.

Line 270-275: I cannot agree with the interpretation of the author. Although Wright (1978) and Hartl & Clark (1997) were cited, these works provide only a general framework. The interpretation of values in empirical studies requires contextual discussion. Moreover, the interpretations in the referred studies do not support the authors’ conclusions. For example, Magnolia species with a global FST of 0.21 and pairwise FST values of 0.03–0.35 were interpreted as showing low to moderate differentiation. In Oreopanax, genetic structure was found among age clusters, but no interpretation of high genetic differentiation was made. Similarly, Fi values of M. schiedeana (0.21–0.28) were interpreted as moderate. Drawing conclusions based solely on arbitrary thresholds, without linking them to the ecological and geographical context, is misleading.

R: We agree and have revised the text accordingly. We now interpret the magnitude of F_{ST}=0.21 in the specific ecological and geographical context of *T. sylvatica*. Where we retain numbers from other TMCF trees, they are used purely as orientation (not as interpretive anchors), acknowledging that qualitative labels vary across taxa, markers, and sampling designs. We emphasize our own AMOVA results (79.5% within; 11.84% among populations) and frame the pattern as moderate spatial structure consistent with an outcrossing tree in discontinuous montane forest. We also corrected language to avoid drawing conclusions on mechanisms that our data does not support.

We appreciate this clarification and agree that our wording conflated local mating with inter-population gene flow. In the revision, we (1) replace “within-population gene flow” with “short-distance pollen dispersal (local mating),” (2) note explicitly that gene flow in the context of F_{ST} refers to exchange among populations, and (3) explain that the combination of local pollen movement and restricted inter-population connectivity across rugged topography can produce high within-population diversity together with non-trivial (but not extreme) among-population differentiation, in line with F_{ST}=0.21 and our AMOVA. We further acknowledge that the observed differentiation likely reflects a balance between limited contemporary movement and occasional inter-population connectivity (e.g., rare bird-mediated seed dispersal) and/or historical connectivity, which can keep F_{ST} from becoming very high. We removed the incorrect phrase “increases individual genetic differentiation within populations.”

Line 275-283: The interpretation that “genetic variation being mostly within populations indicates outcrossing through local, within-population gene flow realized by small insects such as bees” is not logical to me. If gene flow occurred predominantly within populations, one would expect greater differentiation among populations. This point requires clarification.

R: Thank you for pointing this out. We agree that our wording was misleading. Comments on L. 270-283 are discussed above.

Line 282: Add “within population” before “genetic differentiation”. It is otherwise counterintuitive why long-distance seed dispersal by birds would increase overall genetic differentiation.

R: Thank you for this helpful comment. We agree that the wording was ambiguous. We intended to refer to within-population effects of bird-mediated seed dispersal (i.e., increased admixture among individuals

and changes in fine-scale genetic structure), not to an increase in among-population differentiation. We have revised the sentence as suggested.

Line 316-320: This content could be merged with Line 265-269

R: We agree with the referee that although the discussion in the clustering section raises the issue of hybridization, this has indeed been mentioned previously and is repetitive. We have integrated it as suggested, merging both genetic diversity and divergence evidence.

Line 321: The term “metapopulation” is misused in this context. There is no evidence of metapopulation dynamics, and DAPC does not provide information on processes such as local extinction or colonization. I suggest removing this sentence.

R: Of course, without an explicit analysis of connectivity/gene flow, it is too ambitious to use the term metapopulation. We have removed this mention.

Line 330-332: The use of the term “fragmentation” is unclear, as no analysis of landscape fragmentation was conducted. The authors may wish to specify the intended meaning to avoid misunderstanding. In addition, the interpretation of “limited gene flow” and “inbreeding depression” is not supported by the results. Inbreeding depression is a consequence of inbreeding; based on the data, the evidence supports a certain degree of inbreeding but not inbreeding depression. I suggest revising this sentence to: “limited gene flow between northern and southern distribution areas” and “show a certain degree of inbreeding.”

R: We agree that "fragmentation" must be formally assessed using genetic or landscape ecology methods, whereas “gene flow” requires, at least, F_{ST}-based methods; aspects that we have certainly not addressed in this research. Instead, we will refer to the historical "discontinuity" of the SMOr forests in the sense of what is known about historical changes during the Pleistocene. Regarding inbreeding depression, we fully agree that the term refers to the adaptive consequence of inbreeding. We are limiting the wording to inbreeding only.

Reviewer #2: Dear Authors,

Thank you for your great work. All my comments have been addressed except one; to add a paragraph to the Introduction, describing scope of the study and the gap of knowledge. Otherwise, the MS has a good quality.

R: We thank the reviewer for the suggestion as well as for verifying our previous rebuttal. We have modified the final Introduction paragraph into a short, more explicit section that states the knowledge gap and the scope of our study.
