# Supplementary material for: Spatial genetic diversity and populational differentiation of Ternstroemia sylvatica (Ericales: Pentaphylacaceae) in eastern Mexico
Source: PLoS One. 2025 Dec 26;20(12):e0339000. doi: 10.1371/journal.pone.0339000 (PMC12742792; doi:10.1371/journal.pone.0339000)
Supplement: Attachment — Submitted filename: Rebuttal _.pdf [file pone.0339000.s008.pdf]

Rebuttal letter on PONE-D-25-08656 Spatial genetic diversity and populational differentiation of *Ternstroemia sylvatica* (Ericales: Pentaphylacaceae) in eastern Mexico

28 November, 2025.

Dr. Vicente Martínez López
Academic Editor, PLOS ONE

Dear Dr. Martínez López:

We sincerely thank you and the reviewer for the careful evaluation and the continued attention given to the previous versions of our manuscript. We have revised the paper thoroughly in response to the remaining comments from Reviewer 1. As these comments required only minor adjustments, the scientific content of the manuscript remains unchanged, and no modifications were made to the reference list.

All revisions addressing Reviewer 1's feedback are highlighted in the file with tracked changes (*track_changes.docx*). A detailed, point-by-point response to each observation is provided following this letter.

Finally, we would like to express our continued commitment to PLOS ONE's editorial process. We were recently informed that the transformative agreement between our institution (Universidad Nacional Autónoma de México) and PLOS ONE will conclude this year, which may entail additional publication costs beyond our original expectations.

Sincerely,

Dr. Gerardo Rivas
(Corresponding author)

Reviewer #1: The manuscript has been substantially improved in both clarity and overall quality. I have the following minor issues that should be addressed.

Since the revised version does not include line numbers, I have added them, starting from the Abstract (excluding empty lines and tables) so it can be more easily followed.

R: We thank the reviewer for the detailed review that has helped improve the MS. We especially acknowledge the follow-up the reviewer has done throughout the review of the work.

Line 71: mark also the SGSP gap and MB gap in the map (Figure 2)

R: We have included the SGSP and MB gaps labels and bars as they were in previous versions.

Line 206, Line 239: missing “)”.

R: We have applied this change

Line 224-226, Line 263-265, Lines 280-283: these sentences do not convey any meaningful information. Please revise or consider delete.

R: L. 224-226: We agree that the wording was vague, so we modified it to highlight the difficulty in discussing our diversity estimations, given the nature of the other studied species. Finally, we redirected this section to the discussion of intrapopulation genetic variation.

L. 263-265: Yes, it might be risky to support that claim without a broader discussion, so we will omit it.

L. 280-283: We agree that this section is superfluous, so we are removing it.

Line 232: if the sampling scheme has already been exhausted, I doubt that suggesting a denser sampling makes sense. Please revise.

R: We agree. We removed the reference to the sampling scheme.
Line 267: name the tree species

R: We have included the names.

Lines 284-288: is this part repetitive to the content from lines 265-269? Also, name the species in the citation 44. If there are not repetitive, consider combine these two parts together.

R: Yes, we removed the contents of L. 265-269 and retained the wording that is directly related to the AMOVA results.

Line 288: delete “within-population”.

R: We have applied this change.
