# Supplementary material for: Spatial genetic diversity and populational differentiation of Ternstroemia sylvatica (Ericales: Pentaphylacaceae) in eastern Mexico
Source: PLoS One. 2025 Dec 26;20(12):e0339000. doi: 10.1371/journal.pone.0339000 (PMC12742792; doi:10.1371/journal.pone.0339000)
Supplement: Attachment — Submitted filename: Review_PONE-D-25-08656.docx [file pone.0339000.s002.docx]

This study investigated the spatial patterns of population genetic diversity and differentiation of a typical cloud forest tree species *Ternstroemia sylvatica* in eastern Mexico. Using microsatellite markers, the authors estimated genetic indices and applied STRUCTURE and DAPC analysis to identify different genetic clusters. These clusters appear to be explained by geographic separation and local hybridization. Before this study can be published for a broader audience, I have the following thoughts that the authors should consider.

Major comments:

1) **Introduction:**

The goal of this study was not very clearly stated in the introduction. If the primary aim was to present the distribution of population genetic diversity and differentiation of the species, the broader relevance and appeal to a wider scientific audience may be limited. The author should more explicitly articulate the state of the art the study addresses. If the study aims to test the hypothesis that geographical patterns have shaped the genetic structure, e.g. SGSP and MB, this hypothesis should be clearly formulated in the beginning. Additionally, fragmentation is mentioned both in the introduction and later, yet the definition is unclear. What is the difference between fragmentation and biogeographical barrier in the manuscript?

2) **Methodology:**

the AMOVA was not stated in the Methods section. Moreover, the terminology (region, group?) and the interpreted based on this need to be more carefully addressed through out the manuscript.

3) **Interpretation of the results:**

When interpreting the results, the manuscript would benefit from including statistical analysis and adding references or comparisons with other studies or species. If no studies were found, theoretical work should provide a frame. This would help to substantiate the claims made based on the observed values (see below for specific points). Several studies were briefly mentioned in the introduction, however without relationship to the result of the current study.

4) **Discussion structure:**

The structure of the discussion should be improved. Under the first subtitle, the content is not well structured. While the second and last paragraph contain some repetition to the content in the first subtitle and partly disconnected.

5) **Conclusion:**

After reading the manuscript, I find the conclusion that that species exhibits generally general high genetic differentiation is not fully supported. Most of the genetic variance is found within population not between them. The interpretation of inbreeding depression should be first properly addressed in discussion by comparison with other studies. The last paragraph regarding conservation implementation feels disconnected and relies heavily on general statements without sufficient support from the findings. I recommend that the authors focus more closely on summarizing the main results and their direct implications, and avoid broader claims that are not firmly grounded in the data.

Minor points:

Line 41: „land change“, do you mean landscape/land-use change? historical climate change is not “land change”.

Line 43: this sentence seems very abrupt and misplaced.

Line 60: incomplete sentence. A positive relationship between genetic diversity and population size?

Line 61: please be more specific what kind of fragmentation effects are referred to?

Line 62: “Following this author...” is unclear. It would be helpful if the author explicitly stated the main message or findings of the cited publication. Additionally, the knowledge gap that motivated the current study should be clearly articulated in relation to the previous work. This would help the reader better understand the rationale and significance of the study.

Line 76: two populations are missing in the Fig 1

Line 105: “e” should be deleted

Line 118: the acronym ”VOVNI”/”OVNI” is not consistent across the manuscript. Please check.

Line 136: the AMOVA was not mentioned in the Methods.

Line 152: “n” should be “N”; “number fo individuals” should be “number of individuals

Table 1: the number of sampled individuals in each population is very different. And what about the (estimated) population size? This could be a source for the difference in genetic measures, especially allelic richness. Please justify.

Table 3: The terms “region” and “samples” are unclear. Please define them clearly. Also, the AMOVA should be fully described in the Methods section.

Table 3: please align the columns in the table.

Line 182: “MINAS + ACA” should be “MINAS + ELO”?

Line 208: All numbers should be reported with two decimal places to align with the formatting used in Table 2.

Line 207-Line 208: please provide a statistic test (e.g. t-test) to support the claim that “the northern group has a lower expected heterozygosity (He) than the southern group”.

Line 210: The interpretation of “genetic erosion” based simply on the He value is too abstract. Suggest providing additional evidence or set in comparison of other studies.

Line 210-212: Figure 7 and related text should be put into Results section.

Line 218: what is “PAE” analysis?

Line 225: The phrase “values of He about Ho” is unclear.

Line 225: are the values of Fis of 0.42 and 0.38 considered very high? The authors should provide some references or a theoretical framework to make the interpretation of “inbreeding depression”.

Line 233: is Fst=0.205 very high? The author should contextualize this result by comparing it with other studies. Additionally, the logic of the paragraph needs clarification. If the argument is that insect pollination should result in a lower genetic differentiation, it should be noted that the whole studied area covered hundreds of kilometres which is likely beyond typical insect dispersal distances, if not stated specially.

Line 234: It is unclear what is meant by “two main regions.” Please specify which regions are being referred to.

Line 238: “bitt” should be removed.

Line 241-243: the word “species” is missing after “TCMF”. It is also unclear whether a high among-population structure is expected? If so, how high and why? This was not so clear to me. And what does the author mean by “ecological niche”? pollination? Seed dispersal? Local adaptation? Micro-climate? Specific definition is needed.

Line 246-247: was the region differentiation applied between “northern group” and “southern group”? then why would MB gap explain this? MB gap is within the northern group, isn’t it?

Line 248: what is “first-level genetic structure”? K=2? please be consistent

Line 259: “first-level genetic structure” see comment above.

Line 260-262: this part of interpretation could be integrated into Line 228-229.and what does the region here specifically refer to?

Line 266-267” what does the author mean “increasing fragmentation”? what does fragmentation refer to?

Line 271-Line 277: this paragraph is not relevant to the current manuscript.

Line 278-284: this paragraph could be placed after Line 251.both discussing biogeographic barrier.
