# Supplementary material for: Spatial genetic diversity and populational differentiation of Ternstroemia sylvatica (Ericales: Pentaphylacaceae) in eastern Mexico
Source: PLoS One. 2025 Dec 26;20(12):e0339000. doi: 10.1371/journal.pone.0339000 (PMC12742792; doi:10.1371/journal.pone.0339000)
Supplement: Attachment — Submitted filename: Review of manuscript_PLOS One.docx [file pone.0339000.s003.docx]

**Review of manuscript “Spatial genetic diversity and populational differentiation of Ternstroemia sylvatica (Ericales: Pentaphylacaceae) in eastern Mexico”**

**Introduction:**

The first paragraph is directly describing the species, while to reach a wider audience it could be about the type of question that is going to be explored. For instance, it could be a paragraph about the statement in line 61 ‘[literature survey showing that] fragmentation affects the genetic diversity and future conservation of tree species’

Line 60: what does positive mean?

Line 61: Maybe spell out the first author’s name? e.g., Wehenkel et al. (2017) demonstrated…

Lines 66-67: a bit unclear question. Please try to re-phrase it.

Would commenting/advising about conservation plans for this species be also an aim?

**Methods:**

Is it known that the species is diploid? If no, how do you think this may affect the results?

Lines 71-72: add dates of collection. How many samples are collected per population? It is interesting that all material is fresh, and no herbarium material is used.

Line 73: grinded or ground

Line 76: *S. sylvatica* should be *T. sylvatica*

Line 97: please add the reference after each package name.

Line 104: why is K=1 not used?

Line 105: there’s an extra ‘e’ after the reference.

Line 109: please add reference after QGIS 3.22.4

**Results:**

I suggest adding a picture of the species.

Private alleles can be reported for each population.

Fst value between the northern and southern populations can be reported (based on genetic clustering).

Line 139, 143 and 151: pairwise Fst

**Discussion**:

Do these plants grow vegetatively too? Can it affect the genetic structure? And after how long of fragmentation, genetic effects would be observed?

Line 238: an extra ‘bitt’ after reference.

Line 241: add a space before ‘where’

Lines 242-243 gene flow instead of genetic flow

Lines 303-end: using private alleles at each population affects conservation action plans. You may consider it.
