# Supplementary material for: Spatial genetic diversity and populational differentiation of Ternstroemia sylvatica (Ericales: Pentaphylacaceae) in eastern Mexico
Source: PLoS One. 2025 Dec 26;20(12):e0339000. doi: 10.1371/journal.pone.0339000 (PMC12742792; doi:10.1371/journal.pone.0339000)
Supplement: Attachment — Submitted filename: Review.docx [file pone.0339000.s005.docx]

**General comments**: The manuscript has undergone substantial improvement and the overall message and aims of the study are now clearer. However, before it can be considered for publication, the main concern remains the first aim: evaluating genetic diversity and differentiation. I strongly encourage the authors to remain closely aligned with their data and to use appropriately comparable studies to support their statements. Please avoid overstatements and ensure precise and careful use of terms such as “genetic erosion”, “inbreeding depression”.

The structure of the discussion has improved considerably, but I recommend further effort to reconsider the placement of some content, organizing it around the main messages rather than the workflow, while also avoiding repetition.

Please also adjust the abstract accordingly.

Specific points are detailed below.

Line 49: The region of Sierra Gorda may need some explanation. I suggest providing a map (or adding to the existing map) of the relevant geographical and/or administrative units and adding this information in the Table 1 for each sampled population. It would greatly improve readability for readers unfamiliar with the region, particularly in the later sections when the relationship between population genetic pattern and geographical pattern is discussed.

Line 56: The meaning of “have not been evaluated” is unclear. Please clarify what the author intends to convey here? Dose this imply that the aspect is highly relevant but has not yet been assessed, or that it is not relevant in this context.

Lines 65–70: This sentence is overly long and dense, making it difficult to follow. I suggest breaking it into two or more sentences and clarifying the main points. In addition, the broad generalization (“have demonstrated isolation by distance…, low to moderate genetic diversity…”) may be problematic, as not all of the cited studies necessarily report all of these patterns. Please ensure that the summary accurately reflects the findings of each reference.

Line 74: “resulting” should be “resulted”

Line 75: The phrase “land-change use to …” is unclear. Suggest revising to “land-use change due to agriculture and cattle raising” for clarity.

Line 81-82: “Moreover, the genus….of its genetic variation” is repetitive with the Line 79-80: “the lack of knowledge… genetic diversity”. Suggest merging or removing.

Table 1: The Acronym “Tepe” should be “TEPE”.

Line 129: The settings of the DAPC should be reported in detail. Please specify the discrimination functions and the number of principal components used.

Line 132: The methods state that the definitions of “northern group” and “southern group” are based on the first-level STRUCUTRE analysis, but the details of which populations are included are provided at the end of the result. This makes the results difficult to follow. Suggest adding this information already here or at least proving a clear reference to where it is given.

Line 142: “Poliploidy” should be “polyploidy”.

Line 163: “higher” should be “lower”. In addition, why there are no comparisons of *H*_o_ and *A*_r_ (*A*_R_)? If both measures have been reported and the intention is to compare genetic diversity between groups, or state that the genetic diversity is generally lower in the north than in the south, why is only *H*_e_ considered? Suggest either omitting *A*_r_ and *H*_o_ or state the rationale for focusing on only one measure.

Figure 2, 3 and 4: Acronym “OVNI” should be “VOVNI”

Line 201: “hereafter referred to as the northern group” is incorrect as “northern group” was already used before in Methods and Results. Suggest change to “referred to as “northern group” throughout the study”. The same applies to “southern group”. See also comment at Line 132.

Line 225: “MAD” should be “PAMO” I assume.

Line 241: Please clarify why two numbers are reported when only one species is considered—are these values derived from different studies? In addition, the corresponding reference is missing and should be provided.

Line 241-Line 243: For readability, consider combining into a single sentence. For example: “It is comparable to *T. lineate* (*H*_e_=…), which is similar in ecological terms (temperate forest dwellers), has similar fragmentation issues and was evaluated using the same microsatellite set.” However, note that *T. sylvatica* was stated as a tropical instead of temperate species (Line 41), so this statement may need revision.

Line 245: Please standardize the reporting of values to two decimal places throughout the manuscript.

Line 249: The reported *H_e_* of *A. guatemalensis* is below 0.1, whereas the *H*_e_ of Magnolia species was interpreted as media to high. In pine, *H*_e_ is mostly related to population size but not isolation. Therefore, the comparison with the cited studies may not be appropriate. Consequently, the conclusion that *T. sylvatica* suffers from genetic erosion is not fully supported and should be revised. Also, the explanation the low diversity using attribution to population isolation at this place is arbitrary.

Nevertheless, the small population size in the current study, only about 20 individuals per population and all available adults were sampled, could be a good explanation for the low *H*_e_, if the author insists to interpret as such.

Line 250-260: The MB gap falls within the region of “northern group”. If the authors wish to state this subdivision, other clearly defined terms other than “northern, low-diversity group” and “southern, high-diversity group” should be used as this conflict with the defined terms in Line 201-202. One idea could be to report this subdivision in the results (Line 222-Line 228).

Hidalgo should be at least shown on the map so that the referred populations are clear (see also the comment at Line 49). In addition, the content here seems misplaced. I suggest moving this section together with Lines 293-307, as both address this biogeographical pattern.

Line 264: “notorious” change to “noteworthy”

Line 264: Specify which measure was considered for this statement “high diversity spot”.

Line 270-275: I cannot agree with the interpretation of the author. Although Wright (1978) and Hartl & Clark (1997) were cited, these works provide only a general framework. The interpretation of values in empirical studies requires contextual discussion. Moreover, the interpretations in the referred studies do not support the authors’ conclusions. For example, *Magnolia* species with a global *F*_ST_ of 0.21 and pairwise *F*_ST_ values of 0.03–0.35 were interpreted as showing low to moderate differentiation. In *Oreopanax*, genetic structure was found among age clusters, but no interpretation of high genetic differentiation was made. Similarly, *F*_i_ values of *M. schiedeana* (0.21–0.28) were interpreted as moderate. Drawing conclusions based solely on arbitrary thresholds, without linking them to the ecological and geographical context, is misleading.

Line 275-283: The interpretation that “genetic variation being mostly within populations indicates outcrossing through local, within-population gene flow realized by small insects such as bees” is not logical to me. If gene flow occurred predominantly within populations, one would expect greater differentiation among populations. This point requires clarification.

Line 282: Add “within population” before “genetic differentiation”. It is otherwise counterintuitive why long-distance seed dispersal by birds would increase overall genetic differentiation.

Line 316-320: This content could be merged with Line 265-269

Line 321: The term “metapopulation” is misused in this context. There is no evidence of metapopulation dynamics, and DAPC does not provide information on processes such as local extinction or colonization. I suggest removing this sentence.

Line 330-332: The use of the term “fragmentation” is unclear, as no analysis of landscape fragmentation was conducted. The authors may wish to specify the intended meaning to avoid misunderstanding. In addition, the interpretation of “limited gene flow” and “inbreeding depression” is not supported by the results. Inbreeding depression is a consequence of inbreeding; based on the data, the evidence supports a certain degree of inbreeding but not inbreeding depression. I suggest revising this sentence to: “limited gene flow between northern and southern distribution areas” and “show a certain degree of inbreeding.”
