# Supplementary material for: Spatial genetic diversity and populational differentiation of Ternstroemia sylvatica (Ericales: Pentaphylacaceae) in eastern Mexico
Source: PLoS One. 2025 Dec 26;20(12):e0339000. doi: 10.1371/journal.pone.0339000 (PMC12742792; doi:10.1371/journal.pone.0339000)
Supplement: Attachment — Submitted filename: Reviewer_report.docx [file pone.0339000.s007.docx]

The manuscript has been substantially improved in both clarity and overall quality. I have the following minor issues that should be addressed.

Since the revised version does not include line numbers, I have added them, starting from the Abstract (excluding empty lines and tables) so it can be more easily followed.

Line 71: mark also the SGSP gap and MB gap in the map (Figure 2)

Line 206, Line 239: missing “)”.

Line 224-226, Line 263-265, Lines 280-283: these sentences do not convey any meaningful information. Please revise or consider delete.

Line 232: if the sampling scheme has already been exhausted, I doubt that suggesting a denser sampling makes sense. Please revise.

Line 267: name the tree species

Lines284-288: is this part repetitive to the content from lines 265-269? Also, name the species in the citation 44. If there are not repetitive, consider combine these two parts together.

Line 288: delete “within-population”.
